# Chronic diarrhea, eosinophilic ascites, acute pancreatitis and deep venous thrombosis: A case report

**Published:** 2014

**Authors:** Khalid Javid Bhat, Sanjay Bhat, Kalyan Dutt, Sakul Gupta, Hamaad Jeelani Samoon

**Affiliations:** 1 Department of Medicine, ASCOMS & Hospital, Sidhra, Jammu, Jammu & Kashmir, 180017, India.; 2 Department of Internal Medicine, Sir Ganga Ram Hospital, New Delhi, 110060. India.

**Keywords:** Eosinophilic gastroenteritis, Acute pancreatitis, Deep vein thrombosis

## Abstract

***Background: ***Eosinophilic gastroenteritis (EG) is rare and is characterized by recurrent eosinophilic infiltration of the gastrointestinal tract and chronic diarrhea. In this report we present a case of EG with acute pancreatitis and deep vein thrombosis (DVT).

**Case presentation:** A 30 years old male was admitted to our hospital with the complaints of epigastric pain, vomitting and swelling of his left limb for the past six days. He was also having diarrhea for the last several months. He had been evaluated for chronic diarrhea and ascites before he sought the current consultation. Duplex color doppler of left limb showed DVT of distal calf vein. Contrast enhanced CT imaging of abdomen revealed thickening of duodenum, proximal jejunal wall and presence of ascites. Duodenal biopsy showed normal villous pattern with mild inflammation and eosinophilic infiltration. The constellation of clinical presentation, hypereosinophilia, CT and biopsy findings all is in consistence to EG. The patient was treated with prednisolone 20 mg/day for four weeks and tapered slowly. Acute pancreatitis was managed conservatively while DVT was treated with heparin and oral anticoagulants. The patient’s diarrhea settled and ascites resolved completely. At follow up, the absolute eosinophil count was 300/μl and the patient was doing well.

***Conclusion: ***This case report emphasizes that one should consider these rare disorders during the differential diagnosis of unexplained gastrointestinal symptoms in the presence of hypereosinophilia.

Eosinophilic gastroenteritis (EGE), a rare disease is characterized by recurrent eosinophilic infiltration of the gastrointestinal (GI) tract leading to nonspecific GI symptoms that are usually associated with peripheral eosinophilia. It is one of the rare cause of chronic diarrhea (1). Hypereosinophilic syndrome (HES) applies to all clinical presentations in which hypereosinophilia (HE) is directly linked to tissue damage, regardless of whether he can be ascribed to a reactive process, neoplastic process, or another underlying disease (2).There are three subtypes of EGE, mucosal, muscular, and subserosal. However, the mucosal form is the most common (3). Clinical manifestations range from non-specific gastrointestinal complaints to more specific symptoms such as protein-losing enteropathy, luminal obstruction and eosinophilic ascites. Peripheral eosinophilia is common in all subtypes of EGE and is noted in 60-80% of the patients (1). Endoscopic findings may be nonspecific and can range from erythema, friability to erosions. 

Biopsy is highly suggestive of EGE but diagnosis can be missed in up to 25% of cases (4). Moreover, in cases where the diagnosis remains uncertain, CT imaging can help in localizing areas of thickened bowel (5).

We report a case of chronic diarrhea and eosinophilic ascites, an uncommon presentration of EGE which was complicated by acute pancreatitis and deep vein thrombosis (DVT) of left lower limb simultaneously, a rare clinical association. 

## Case Report

**Figure 1:**
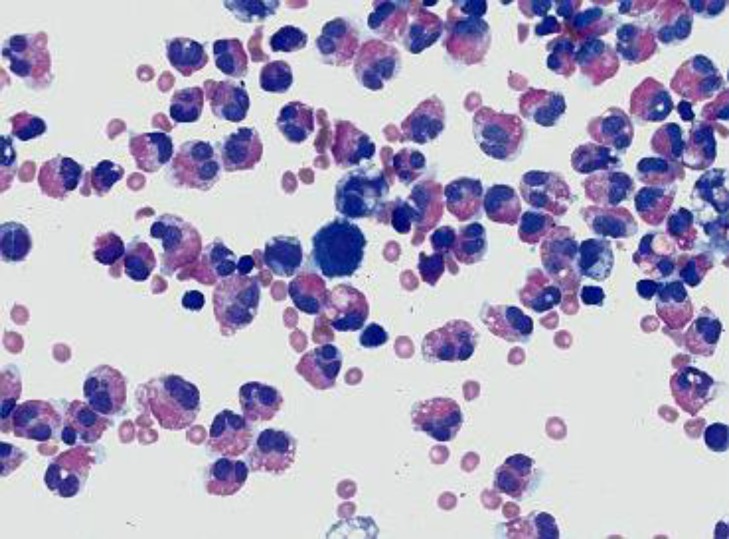
Ascitic fluid showing increased numbers of eosinophils with Wright-Giemsa stain (magnification x 400

**Figure 2:**
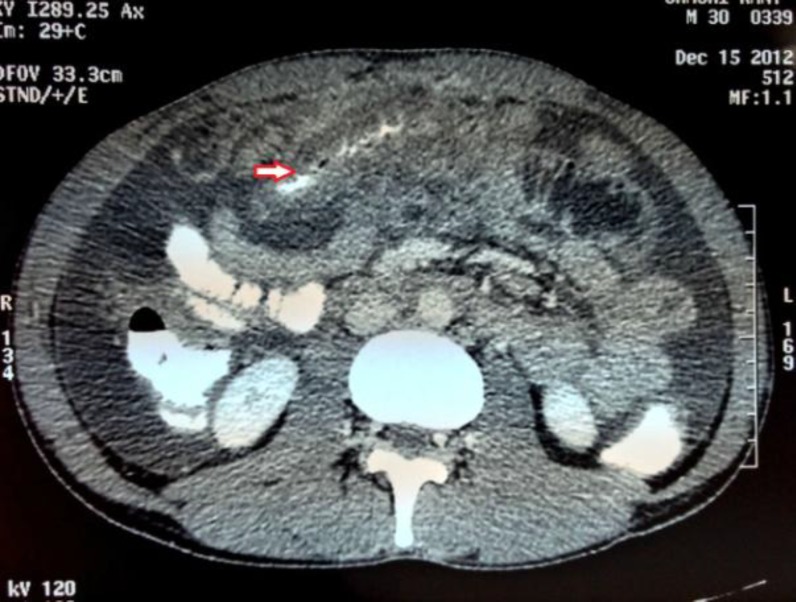
Contrast enhanced CT imaging of abdomen showing intestinal thickening of small gut (jujenum) and ascites

**Figure 3:**
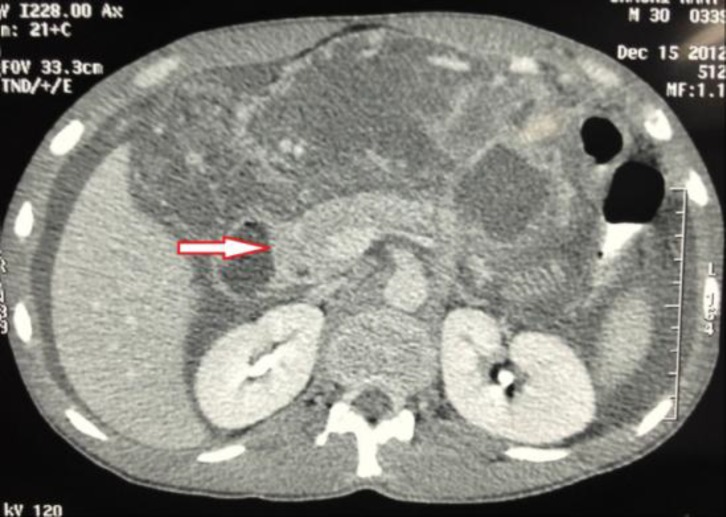
Contrast enhanced CT imaging of abdomen showing mild edematous pancreas.

A 30-year old male was admitted to our hospital with the complaints of epigastric pain, vomitting and swelling of his left limb for past six days. He was also having diarrhea for the last several months. Detailed history revealed that he had been reasonably evaluated for chronic diarrhea and ascites for the last six months before he sought the current consultation. There was no history of fever, jaundice, blood in the stool or any bladder symptoms. He was non- alcoholic and had no personal or family history of any allergy or asthma. On admission, the patient looked emaciated with dry skin, had oral temperature of 98 ^0^ F, pulse - 90 beats/min and blood pressure of 130/90 mmHg. Abdominal examination revealed periumbilical tenderness with appreciable ascites and there were no signs suggestive of chronic liver disease. He had edematous and tender swelling of his left leg with limited passive mobility. His peripheral pulses were normal. Laboratory investigations showed hemoglobin -10.7 g/dL , total leukocyte count - 10,800/mm^3^ with predominant eosinophils (22%), absolute eosinophil count - 2,376/mm^3^ (normal range, 0 to 500/ mm^3^) and platelet count - 2.5 lakh/cumm^3^. Serum amylase measured 650U/L (reference range: 0-200 U/L), serum lipase -150 U/L (reference range: 30-110 U/L), serum bilirubin - 1.0 mg/dl, serum albumin - 4.5 mg/dl, aspartate transaminase - 46 IU/L, alanine transaminase - 80 IU/L and alkaline phosphatase - 42 U/L. He was tested negative for HIV-1 and 2 (ELISA), HBsAg and anti-HCV antibody. Routine stool examination and fecal fat estimation of the stool was normal. Ultrasound showed moderate ascites with normal echotexture of the liver and mild oedematous pancreas. Ascitic fluid study revealed high SAAG ascites with total leukocyte count-1000/ mL, 90% of which were eosinophils (figure 1), adenosine deaminase (ADA) - 18 IU/L and amylase - 28U/L ( range, 0 - 100 U/L). Serum IgE level was elevated at 548 IU/mL (normal < 180 IU/mL). Duplex color doppler of left limb showed thrombotic occlusion at the level of distal calf vein suggestive of deep vein thrombosis (DVT). Contrast enhanced CT imaging of abdomen done two days later revealed thickening of duodenum, proximal jejunal wall and presence of ascites (figure 2) with mildly enlarged pancreas (figure 3). 

**Figure 4:**
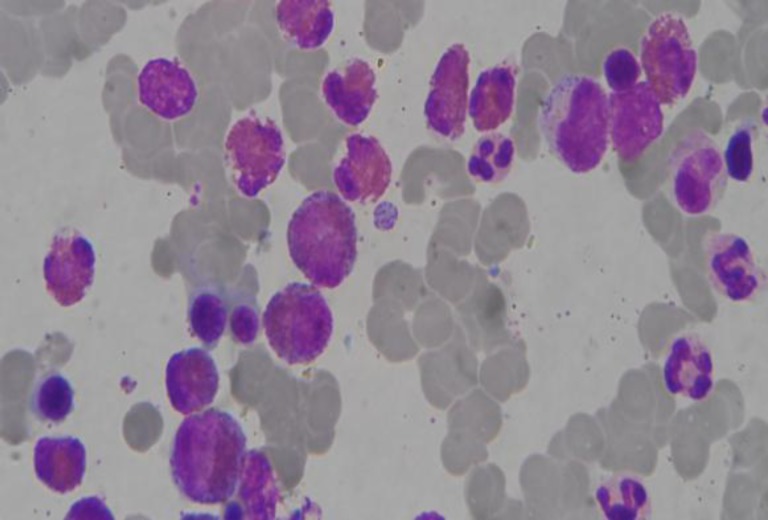
Bonemarrow picture showing markedly increased number of eosinophils. (Leishman stain × 1000).

Once the patient was stable, upper gastrointestinal endoscopy showed mild hyperemia of antral mucosa of stomach, and duodenum. Duodenal biopsy showed normal villous pattern with mild inflammation and eosinophilic infiltration (12/HPF). To ascertain other causes of HE, bone marrow biopsy was done which revealed erythroid hyperplasia with normoblastic erythropiesis and prominent esonophilic infiltration (25%) (figure 4).

The constellation of clinical presentation, hypereosinophilia, CT and biopsy findings and an avid clinical response to steroids was suggestive of EGE. Acute pancreatitis was managed conservatively while DVT was treated with low molecular weight heparin and oral anticoagulants. To treat EGE and manage HE, the patient was put on oral prednisone (20mg/day) for four weeks and tapered slowly. The patient’s diarrhea settled and ascites resolved completely. At follow-up, a repeat peripheral blood count showed a considerable fall in the absolute eosinophil count (300/μl) and the patient was doing well.

## Discussion

Eosinophilic gastrointestinal disorders are exceedingly rare and manifest through all races and ages, from infancy through adulthood (6). In HES, eosinophil-induced organ damage may vary from angioedema, eczema and peripheral neuropathy to cardiac fibrosis and thrombosis. About 25% of cases with HES, however, have eosinophilic infiltration in the gastrointestinal tract and there may be little to distinguish between EGE and HES, especially if the latter is early in the clinical course and restricted to the intestinal mucosa alone (7). 

After a reasonable workup, the cause of diarrhea and eosinophilic ascites in our patient seemed to be EGE which was supported further by the biopsy, CT findings and an avid clinical response to the steroid therapy. Chronic diarrhea and eosinophilic ascites are rare manifestations of EGE (1). Acute pancreatitis has been previously reported in association with EGE (8). However, the unusual complication of acute DVT and acute pancreatitis occurring simultaneously in a patient with EGE makes it a very rare case. Initially, pancreatitis seemed to be of unclear etiology as our patient was non - alcoholic and had no history of any gall bladder disease. Nonetheless, eosoninophil-induced direct toxic endothelial injury or obstruction of the pancreatic duct may explain the cause of acute pancreatitis in our case (9). The mechanism of DVT in our case can be explained by endothelial dysfunction due to the degranulation of activated eosinophils (cationic protein, major basic protein) and inhibition of the natural anticoagulant pathways (thrombomodulin, heparan sulphate, and antithrombin III ) (10).

The diagnosis of eosinophilic gastrointestinal disorders (EGID) should be considered in patients with history of chronic diarrhea, peripheral esonophilia and esonophilic ascites. Evaluation and risk determination for hypereosinophilic syndrome (HES) in patients with apparent EGID is also important. Our case also emphasizes the importance of being aware of the ‘**forme frustes’** of hypereosinophilic syndrome and various complications arising from the same.
